# Effect of Silane-Containing Adhesives on Repair Bond Strength between Fresh and Aged Composite Materials—A Pilot Study

**DOI:** 10.3390/ma17184646

**Published:** 2024-09-22

**Authors:** Petra Gajski, Matej Par, Zrinka Tarle, Danijela Marovic

**Affiliations:** 1 Dental Clinic Apolonija, 10000 Zagreb, Croatia; pgajski@gmail.com; 2 Department of Endodontics and Restorative Dentistry, University of Zagreb School of Dental Medicine, 10000 Zagreb, Croatia; mpar@sfzg.unizg.hr (M.P.); tarle@sfzg.unizg.hr (Z.T.)

**Keywords:** shear bond strength, composite repair, aging, silane, adhesives

## Abstract

This study investigated the effects of different surface treatments and a silane-containing adhesive on the repair bond strength between fresh and aged resin composites. A total of 140 composite specimens were prepared and aged for 24 h or 4 months. Each group was subdivided into seven subgroups (*n* = 10) depending on the surface treatment (no surface treatment (NT), sandblasting (SAND), or Sof-lex coarse disc (DISC)) in combination with the use of the silane-containing adhesive ScotchBond Universal Plus (SBU) or an adhesive without silane Prime&Bond Universal (P&B). The same composite was used for the repair as for the primary specimen. Specimens were dark stored in distilled water at 37 °C for 28 days. Shear bond strength was tested at a crosshead speed of 0.5 mm/min. The Kruskal–Wallis test with Bonferroni’s post-hoc adjustment (α = 0.05) and the Mann–Whitney U-test were used for the statistical analysis. The results are shown as the median with the interquartile range. The highest bond strength (MPa) was achieved after 24 h in the DISC+P&B (20.39(16.85–28.83)). In the fresh 24 h group, the SAND+P&B (12.25(8.28–15.05)) and DISC+SBU (18.37(15.16–21.29)) were statistically similar. In the 4-month groups, both adhesives and surface treatments performed similarly. The NT, SAND, and DISC groups without adhesives had the lowest bond strength. In the repair of fresh or aged composite, the silane-containing adhesive SBU was not superior to the adhesive without the silane (P&B).

## 1. Introduction

With the improvement of bonding, curing, and resin systems, the use of resin-based composite materials in restorative dentistry has become routine for direct restorations [1]. Nevertheless, dentists spend 70% of their chairside time replacing restorations due to secondary caries, fractures, colour changes, inadequate colour matching with the surrounding tooth substrate, insufficient contour, or spacing at the proximal surface. Some errors may occur and can be detected immediately after placement of the filling or later after a short period of time [2]. The treatment choice is either a complete replacement or a repair of the existing restoration. Complete removal of the composite restoration is not desirable, as it is difficult to limit the removal to the composite restoration without damaging the adjacent healthy tooth structure and without creating wider preparations. Once a tooth is restored, a continuous process of replacement of restorations is triggered, eventually leading to tooth loss after several cycles, a phenomenon known as the “restorative cycle” or “death spiral” [3,4,5,6]. For this reason, minimally invasive dentistry favours the repair rather than the replacement of defective restorations [7].

The bond between two composite layers placed immediately one after the other is achieved through an oxygen-inhibited layer of unpolymerised resin [4,5]. However, if the composite has been contaminated, polished, or aged, the bond with the new composite cannot be adequately established due to the lack of an intact oxygen-inhibited layer [8,9]. Several techniques have been proposed, employing macro and micromechanical retention [10]. Macromechanical retention involves creating holes, undercuts, or roughening the surface with a coarse bur. For micromechanical roughening of the surface, acid etching (hydrofluoric or phosphoric acid) or air abrasion have been suggested, followed by silane application in a separate step and the use of adhesive prior to the application of a new composite layer. In addition to sandblasting, surface abrasion with discs is also used to achieve micromechanical retention for better bonding results [11].

Silane is a coupling agent used to create the siloxane bond with the silica-based structures (glass fillers) on one side and the carbon–carbon bond with the methacrylates from the resin matrix on the other [12]. It is not only used for the filler/matrix bond in composites but is also considered an essential bonding agent for the cementation of silica-based materials or silica-coated materials for indirect restorations [13]. For this purpose, commercial products are available in a separate bottle.

With the advent of new silane-containing adhesive formulations, the question arises as to whether the repair procedure could be simplified by using these adhesives without a separate silane application. Currently, there are no official guidelines for the use of silane-containing adhesives in composite repair. A recently published scoping review and other studies investigating this topic [14] brought inconclusive results. Some studies found that there was no difference in the performance of a universal adhesive with or without a silane pretreatment on the surface of the aged composite [15,16,17], while others found a significant difference in favour of additional silane pretreatment [18,19,20].

The repair of aged composites is challenging because the aged composite no longer contains free radicals after the initial polymerisation [21,22]. When composites are exposed to an aqueous environment for a prolonged period of time, such as in the oral cavity, water absorption occurs. Water-saturated composites may pose an additional challenge to repair compared to freshly placed composites, which may still contain some radical species. The aim of this study was, therefore, to investigate the effects of different surface treatments and silane-containing adhesive on the repair bond strength between fresh (24 h) or aged (4 months) resin composites.

The null hypotheses tested in this study assumed no difference in repair bond strength:

1. Between fresh and aged resin composites;
2. Among different surface treatments;
3. Without and with the application of silane-containing adhesives or adhesives without silane.

## 2. Materials and Methods

In this study, one composite material and two adhesive systems were used (Table 1). 

Composite specimens (Filtek Supreme Ultra shade: A2B, 3M) were prepared at room temperature (22 ± 1 °C) in discoid Teflon moulds with a diameter of 5 mm and a height of 2 mm. The moulds were placed on a polyethylene (PET) strip, filled with uncured composite, and covered with another PET strip. The samples were light-cured using a Bluephase Style (Bluephase, Ivoclar Vivadent, Schaan, Liechtenstein; irradiance 1000 mW/cm^2^) for 20 s on each side. The upper side of the sample in the no-surface treatment group was levelled with the Optrasculpt instrument (Ivoclar Vivadent) and then light-cured in the same way.

**Figure 1:**
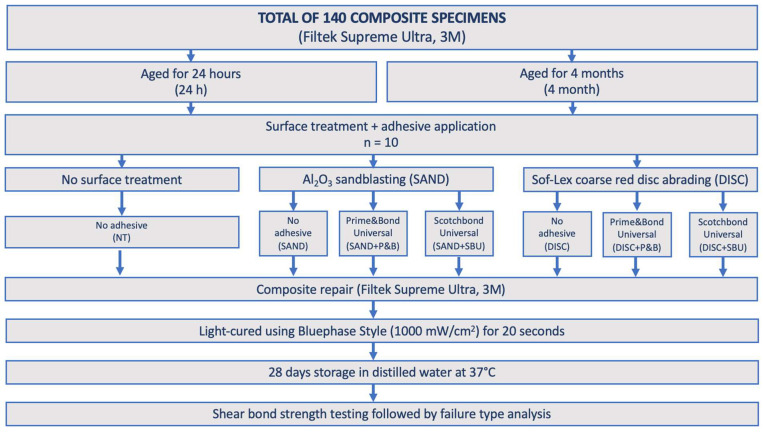
Flow chart of the study.

The flow chart of the experimental procedure is shown in Figure 1.

A total of 140 composite specimens were prepared and dark stored in distilled water at 37 °C for the following times:

(1) 24 h;
(2) 4 months.

Distilled water was changed every 30 days in the 4-month groups.

Each group was subdivided into seven subgroups (*n* = 10) depending on surface treatment before performing repair:

1. No surface treatment (NT);
2. Al_2_O_3_ sandblasting (SAND);
3. Al_2_O_3_ sandblasting + Prime&Bond Universal (SAND+P&B);
4. Al_2_O_3_ sandblasting + Scotchbond Universal Plus (SAND+SBU);
5. Sof-Lex coarse red disc abrading (DISC);
6. Sof-Lex coarse red disc abrading + Prime&Bond Universal (DISC+P&B);
7. Sof-Lex coarse red disc abrading + Scotchbond Universal Plus (DISC+SBU).

In subgroups 2, 3, and 4, sandblasting was performed in a sandblasting unit (Basic classic Fine sandblasting unit, 25–70 µm, 220–240 V, Renfert, Hilzingen, Germany) with 50 μm of Al_2_O_3_ sand (Cobra aluoxid 50 µm, Renfert, Germany). In groups 5, 6, and 7, surfaces were abraded with a Soflex red disc (3M™ Sof-Lex™ Finishing and Polishing Discs–Extra Thin/Coarse, 12.7 mm, 8692C, 3M).

After surface treatment, the silane-containing adhesive Scotchbond Universal (3M) or an adhesive without silane Prime&Bond Universal (Dentsply) was applied for 20 s, gently air dried for 5 s, and light-cured using a Bluephase Style (1000 mW/cm^2^) for 10 s.

**Figure 2:**
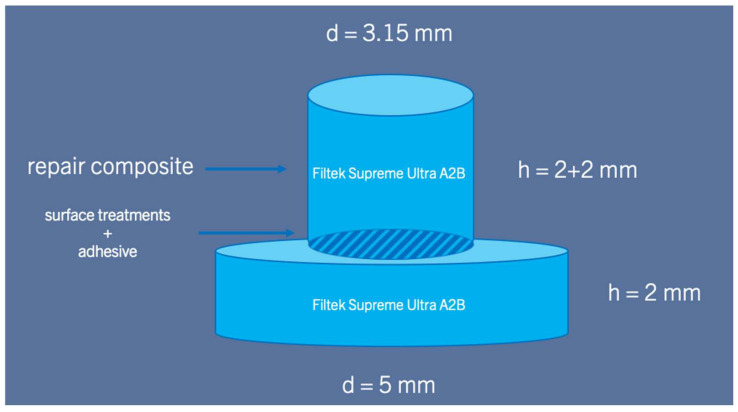
Illustration of the specimen.

The composite used for repair was the same composite used to make the primary specimen, Filtek Supreme Ultra. Repair composite was applied using a cylindrical mould in two layers with a diameter of 3.15 mm and a height of 2 mm. Each layer was light-cured using a Bluephase Style (1000 mW/cm^2^) for 20 s (Figure 2).

Specimens were dark stored in distilled water at 37 °C for 28 days. Shear bond strength (SBS) was tested by loading the specimens in a universal testing machine (Inspekt Duo 5 kN; Hegewald & Peschke, Nossen, Germany) at a constant crosshead speed of 0.5 mm/min until fracture.

SBS was calculated according to the following equation:

SBS (MPa) = *F_max_*(*N*)/*A*; (m^2^)

where *F_max_* denotes the maximum load, and A denotes the bonded surface area.

The fractured surface of each specimen in both the 24 h and 4-month groups was visually examined to determine whether the fracture traversed the bonded interface (denoted as A for “*adhesive fracture*”), went through the lower composite specimen (denoted as C for “*cohesive fracture*”), or the specimen was debonded during the storage in distilled water before testing (denoted as PTF for “*pre-test failure*”).

Bond strength values were compared using nonparametric statistics to address significant departures from normality due to the pre-test failures, which were attributed to zero values. The comparisons among treatments and adhesives were performed using the independent samples Kruskal–Wallis test with Bonferroni’s post-hoc adjustment. The comparisons between 24 h and 4-month groups were performed using independent samples of the Mann–Whitney U-test. Statistical analysis was performed using SPSS 25.0 (IBM, Armonk, NY, USA). The level of significance for all analyses was 0.05. The results are shown as the median and the interquartile range (IQR).

## 3. Results

**Figure 3:**
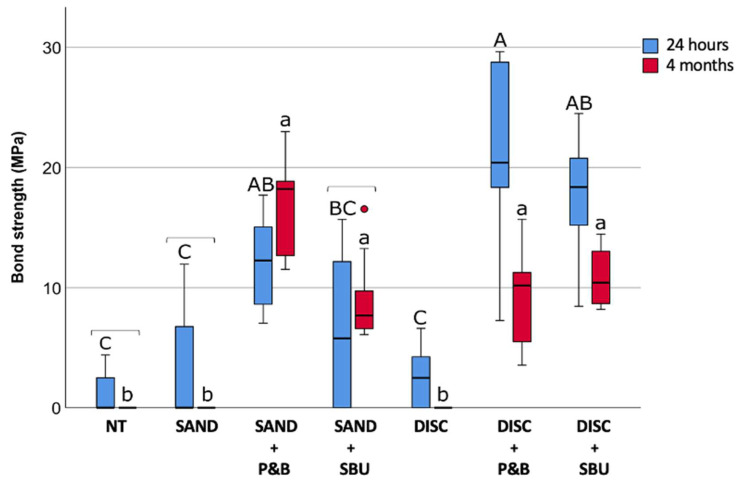
Shear bond strength for tested groups (*n* = 10). Data are presented as medians and interquartile ranges. Identical uppercase letters denote statistically similar values within the 24 h group; identical lowercase letters denote statistically similar values within the 4-month group; square brackets denote statistically similar values for comparisons 24 h vs. 4 months.

The results of the shear bond strength for the 24 h and 4-month-aged specimens are shown in Figure 3.

The treatments without adhesive application, NT, SAND, and DISC, had the lowest values, with no significant differences between them. Only disc-abrasion (DISC) showed slightly, but nonetheless statistically significant, improved SBS for fresh 24 h specimens compared to aged 4-month specimens.

Similarly, for the fresh 24 h specimens, disc-abrasion followed by the application of any of the tested adhesives achieved a higher SBS than sandblasting with adhesives, although not statistically significant. Within the 24 h group, SAND+P&B (12.25(8.28–15.05)) and DISC+SBU (18.37(15.16–21.29)) had statistically similar results. Overall, the highest SBS in the 24 h group was the group with surface treatment DISC+P&B (20.39(16.85–28.83)).

In the 4-month group, there was no statistical difference between sandblasting, disc-abrasion, and any of the tested adhesives. The SBS values of SAND+SBU were statistically similar for comparison between 24 h and 4 months.

**Figure 4:**
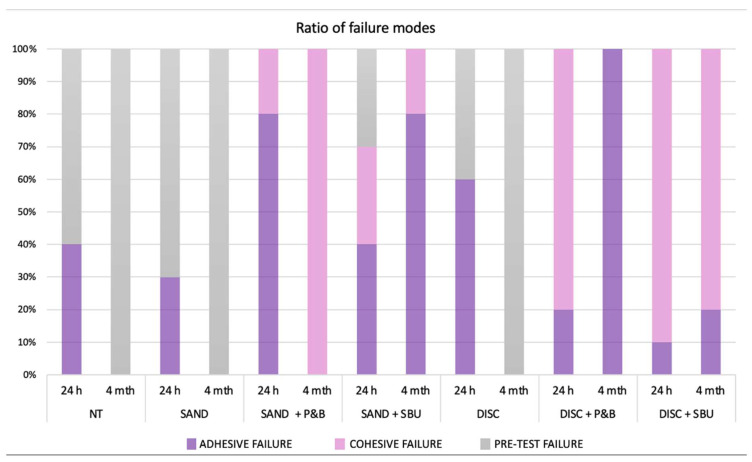
Distribution (percentage) of fracture mode. A: adhesive fracture, C: cohesive fracture, PTF: pre-test failure.

The failure mode of each subgroup in the 24 h and 4-month groups is shown in Figure 4. All specimens in the NT, SAND, and DISC groups showed pre-test failure in the 4-month-aged specimens. The treatment without adhesive application and no treatment group showed only adhesive fractures in the 24 h group. The 4-month DISC+P&B group exhibited the highest adhesive failure ratio, and the 4-month SAND+P&B exhibited the highest cohesive failure ratio among all groups. The treatments with sandblasting in the 24 h group showed more adhesive than cohesive fractures, and the treatments with disc showed more cohesive than adhesive fractures.

## 4. Discussion

The composite repair is affected by many factors, including aging of the restoration in a humid and aqueous environment, high water saturation, surface preparation, and others [23]. In this study, different surface treatments and adhesives were evaluated, and SBS was used to measure the repair bond strength of fresh (24 h) and aged specimens (4 months).

In the present study, no significant difference in the repair bond strength values was found when silane-containing adhesive was used. This result is consistent with other studies [24,25]. In 1997, Brosch et al. showed that the application of silane in a separate step and a 120 s penetration prior to adhesive application had a positive effect on the repair bond strength [5]. More recently, in 2019, Flury et al. compared the use of silane in a separate step and subsequent adhesive application with silane-containing adhesive alone and found no statistically significant difference, neither after 24 h nor after 1 year [26]. There are three different mechanisms in the process of composite repair: chemical bond formation to the matrix, chemical bonds to the exposed filler particles, and micromechanical retention achieved through penetration of the monomer components to micro irregularities in the matrix [1]. However, the published literature shows great diversity regarding the effects of silane-containing adhesives. Some studies showed no significant difference in repair bond strength between the silane-containing adhesive and adhesive without silane [24], and one study showed that the adhesives without silane were more efficient than the silane-containing adhesive [25]. In contrast, Chen et al. demonstrated that silane-containing adhesives improve the bond between composites with surface treatment after 24 h in a 37 °C water bath [4].

It is considered that the surface treatment of the old composite is necessary to achieve bonding between old and new composites [5,8,23,27,28,29]. Sandblasting and abrading the surface with discs are surface treatments that roughen the surface to increase the retentive surface available for bonding aged and fresh composites. Both surface treatments cause “micro” retentive features, while diamond stone or green Carborundum stones cause “macro” and “micro” retentive features, respectively; without a bonding system, greater bond strength is expected to be achieved in “macro” retentive features. On the other hand, with a bonding system, a better wetting of the surface occurs as the adhesive resin infiltrates into the composite microscopic surfaces [5,28,29]. The results of the present study are in agreement with the reports about “micro” and “macro” retentive features [5,14,28,29,30]; the lowest bond strength had groups with surface treatments and without the use of adhesives. The combination of surface treatment and use of the adhesive showed better results in both aged and fresh groups.

A restoration that is repaired in the oral cavity has been aging in a humid environment for some time, water saturation of composite resin has been reached and free radical activity has ended [31]. Absorbed water causes the plastification of the matrix, formation of microcracks, degradation of resin, and debonding of the filler–matrix interfaces. The silane coupling layer at the filler–matrix interface can hydrolyse if exposed to water for a prolonged period of time [13]. As the bond deteriorates, the filler particles may detach or separate from the resin matrix, and the surface of the composite may show signs of erosion or wear due to interaction with water, resulting in changes in surface texture and appearance. Therefore, there is no standardized procedure to simulate the aging of composites in oral conditions [1]. In this study, specimens were dark stored in distilled water at 37 °C, first before the repair, either for 24 h or 4 months, and then after the repair for another 28 days. The aged specimens with a disc surface treatment and the use of adhesives showed lower results than the fresh specimens with the same treatment and the same adhesive. This was not the case for the sandblasted specimens with either of the two adhesives used. The possible answer to this question remains to be seen in future studies.

However, the type of composite used could be responsible for the differences in the surface treatments. In this study, we used the same nanocomposite for both the base and the repair. Filtek Supreme is filled with agglomerated and non-agglomerated nanofillers consisting of 4–20 nm of silica and zirconia oxide particles. According to Mitra et al., Filtek Supreme reacts to surface wear by mechanically breaking off the filler particles, which are present in clusters found with round defects on the surface of the specimen after sandblasting with 25 µm of aluminium oxide particles [30,31]. This surface filler loss could have caused a somewhat lower repair bond strength in the present study for both fresh and aged specimens because of the reduced amount of fillers that could react with adhesive/silane-containing adhesive. On the other hand, there are no data in the literature on the surface roughness of composites achieved by using the red Sof-lex disc only, and we can only assume that the use of the disc did not remove many surface filler particles.

Regardless of these differences, all aged specimens were statistically similar in the groups that received a surface treatment (either disc or sandblasting) and one of the tested adhesives. Under the present experimental conditions, this suggests that any micromechanical surface abrasion prior to adhesive application will have a similar effect on composite repair if the repair is not performed immediately after composite application. The silane-containing adhesive was not a decisive factor in improving the bond strength of the repair in the present study. Further studies on repair bond strength with prolonged exposure to water after repair are needed to determine the long-term effects of the silane-containing versus the silane-free adhesive in composite repair.

An important limitation associated with this study is the high variability of the data, which made it difficult to identify statistically significant differences. The bond strength data are generally known for their high heterogeneity, regardless of whether macro [32] or micro-testing [33,34] methods are used, which reduces statistical power and blurs differences between groups. In addition, a high frequency of pre-test failures resulted in skewed distributions that precluded the use of parametric tests, which further reduced the discriminatory power of the statistical analysis.

## 5. Conclusions

The present study shows that the silane-containing adhesive SBU was not superior to an adhesive without silane (P&B) in the repair of fresh or aged composite. For a successful repair of composite fillings, surface treatment with sandblasting or surface roughening in combination with adhesive is recommended. A purely mechanical pre-treatment without the use of adhesive is not advisable for the repair of composite.

## Figures and Tables

**Table 1:** Resin-based composite and adhesives used in this study.

Materials	Name	Composition	ManufacturerANUFACTURER
Composite	Filtek Supreme Ultra A2B shade	Bis-GMA, UDMA, TEGDMA, bis-EMA(6), filler (78.5 wt%/63.3 vol%)	3M, St. Paul, MN, USA
Adhesive	Prime&Bond Universal	Bi- and multifunctional acrylate, phosphoric acid modified acrylate resin, initiator, stabilizer, isopropanol, water	Dentsply Sirona, Konstanz, Germany
Silane-containing adhesive	ScotchBond Universal Plus	MDP phosphate monomer, HEMA, 3M^TM^ Vitrebond^TM^ Copolymer, filler, Ethanol/water, initiators, **silane**, dual-cure accelerator, dimethacrylate resins containing BisGMA	3M, St. Paul, MN, USA

## Data Availability

Data supporting the reported results are available on request from the corresponding author.
